# *Wisteria floribunda* agglutinin-positive mac-2 binding protein as an age-independent fibrosis marker in nonalcoholic fatty liver disease

**DOI:** 10.1038/s41598-019-46172-1

**Published:** 2019-07-12

**Authors:** Nobuharu Tamaki, Mayu Higuchi, Masayuki Kurosaki, Sakura Kirino, Leona Osawa, Keiya Watakabe, Wan Wang, Mao Okada, Takao Shimizu, Kenta Takaura, Hitomi Takada, Shun Kaneko, Yutaka Yasui, Kaoru Tsuchiya, Hiroyuki Nakanishi, Jun Itakura, Yuka Takahashi, Nobuyuki Enomoto, Namiki Izumi

**Affiliations:** 1 Department of Gastroenterology and Hepatology, Musashino Red Cross Hospital, Tokyo, Japan; 2 First Department of Internal Medicine, Faculty of Medicine, University of Yamanashi, Chuo, Japan

**Keywords:** Liver diseases, Hepatology

## Abstract

The assessment of liver fibrosis is essential because it correlates with mortality risk in nonalcoholic fatty liver disease (NAFLD). This study aims to examine whether serum fibrosis markers could identify candidate patients likely to have advanced fibrosis. We enrolled 352 patients with NAFLD and performed liver biopsies in 97 patients. The area under the receiver operating characteristic curve (AUROC) of liver stiffness by magnetic resonance elastography for histological advanced fibrosis was 0.910, and the optimal cutoff value was 4.07 kPa. To predict severe liver stiffness (≥4.07 kPa), the AUROC for *Wisteria floribunda* agglutinin-positive mac-2 binding protein (WFA^+^-M2BP) and FIB-4 were 0.897 (cutoff value, 1.08) and 0.880 (cutoff value, 2.53), respectively. After stratification of patients into four age groups as quartile, the optimal cutoff values of WFA^+^-M2BP for predicting severe liver stiffness were similar in each group (1.09, 1.08, 1.10, and 1.12). On the other hand, those of FIB-4 increased in parallel with age (1.47, 2.19, 2.99, and 3.88). In conclusion, WFA^+^-M2BP was precise for estimating severe liver stiffness in NAFLD with single cutoff value independent of age. Hence, identifying high-risk cases using WFA^+^-M2BP from a large number of NAFLD patients is clinically significant.

## Introduction

The incidence of nonalcoholic fatty liver disease (NAFLD) is gradually increasing, and >25% of the population globally had NAFLD^1^. Likewise, the incidence of NAFLD has exceeded 20% even in Japan, making it the leading chronic liver disease in the country^2^. Subsequently, the occurrence of liver cirrhosis and hepatocellular carcinoma (HCC) is considered to increase with the growing incidence of NAFLD, and early detection of high-risk patients is clinically essential.

In recent years, liver fibrosis has been reported to correlate with liver-related events and mortality risk in NAFLD, with liver fibrosis being the major prognostic predictor^3–5^. Thus, the precise assessment of liver fibrosis is clinically significant. Although liver biopsies have been used for assessing liver fibrosis^6^, noninvasive methods that could be alternative to liver biopsies have been developed because of complications and sampling errors involved in liver biopsies^7^. Magnetic resonance elastography (MRE) is a noninvasive diagnostic method for liver fibrosis that substitutes liver biopsy^8^. In addition, severe liver stiffness detected using MRE correlates with the HCC development in patients with chronic hepatitis C after viral eradication^9^. Reportedly, the diagnostic accuracy of MRE for fibrosis stage in NAFLD is superior to that using other methods such as serum fibrosis markers or ultrasonic elastography^10,11^. Thus, MRE has also been used as a diagnostic method in clinical trials of therapeutic drugs for NAFLD^12,13^.

It is conceivable to precisely diagnose NAFLD using MRE, and this method could be measured recurrently for monitoring; however, using this method for screening is challenging because of the high cost and availability. Thus, the development of a method that facilitates easy identification of high-risk patients with advanced fibrosis due to NAFLD is imperative^14^, and the construction of an algorithm to identify high-risk patients is an unmet clinical need.

This study aims to validate the diagnostic accuracy of MRE for advanced liver fibrosis and examines whether simple serum fibrosis markers could identify candidate patients who are likely to have advanced fibrosis, among a wide range of NAFLD, for detailed examination by MRE.

## Results

### Patients’ background information

Table 1 presents the patients’ background information. We enrolled 352 patients (average age: 62.2 ± 14.3 years; 170 males and 182 females) in this study. Of all, 48 patients had a history of HCC and these patients were included in the analysis. We performed a liver biopsy in 97 patients, and the fibrosis stages were 1, 2, 3, and 4 in 21, 19, 33, and 24 patients, respectively. The mean liver stiffness was 4.36 ± 2.5 kPa.

**Table 1:** Patients background.

Age (years)	62.2 ± 14.3
Sex (male/female)	170/182
BMI (kg/m^2^)	26.2 ± 4.4
Diabetes (yes/no)	129/223
AST (IU/L)	49.5 ± 31.7
ALT (IU/L)	59.1 ± 47.4
Albumin (g/dl)	4.18 ± 0.58
Platelet counts (×10^4^/μL)	18.8 ± 6.7
Total cholesterol (mg/dL)	193 ± 36.9
Triglycerides (mg/dL)	159 ± 115
Hemoglobin A1c (%)	6.49 ± 1.0
FIB-4	2.78 ± 2.1
WFA^+^-M2BP (COI)	1.59 ± 1.64
Liver stiffness by MRE (kPa)	4.36 ± 2.5
History of HCC (yes/no)	48/304
Brunt stage(1/2/3/4)	21/19/33/24

BMI, body mass index; AST, aspartate aminotransferase; ALT, alanine aminotransferase; WFA^+^-M2BP, wisteria floribunda agglutinin-positive mac-2 binding protein; MRE, magnetic resonanse elastography; HCC, hepatocellular carcinoma.

### Correlation between fibrosis stage and liver stiffness

**Figure 1:**
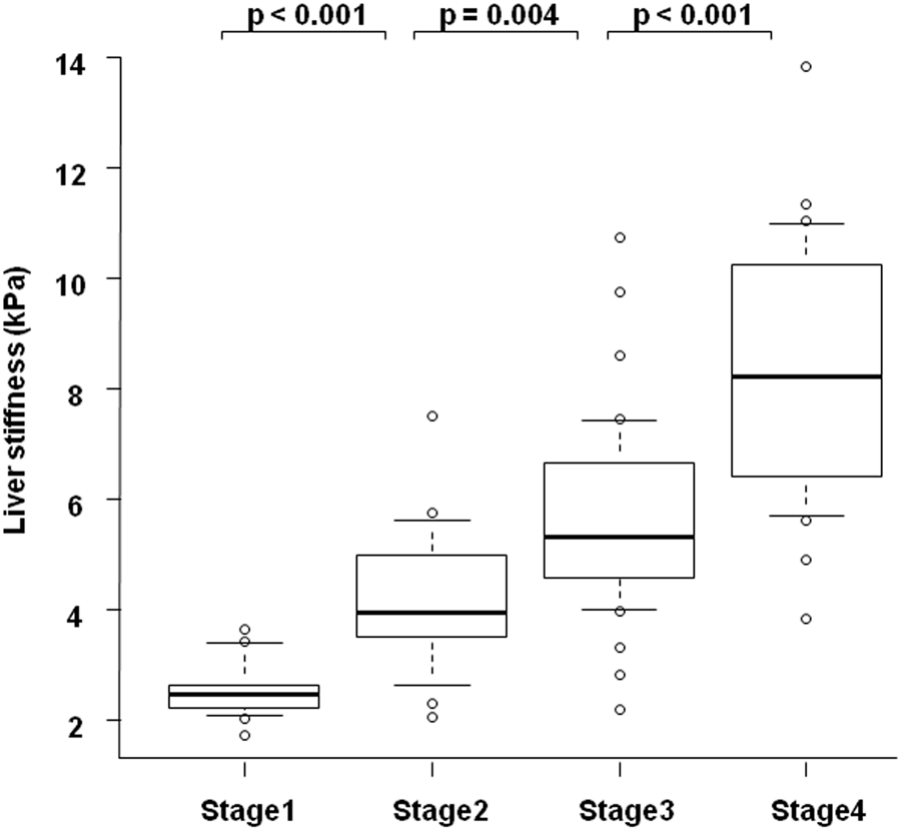
The correlation between liver stiffness measured by magnetic resonance elastography (MRE) and histological fibrosis stage. The boxplot of liver stiffness was shown according to each fibrosis stage. The bottom and top of each box represent the 25^th^ and 75^th^ percentiles, giving the interquartile range. The line through the box indicates the median value, and the error bar indicates 10^th^ and 90^th^ percentiles.

We examined the correlation between the fibrosis stage and liver stiffness. The median liver stiffness values (minimum–maximum) for patients in each stage (1, 2, 3, and 4) were 2.48 (1.72–3.64), 3.94 (2.05–7.52), 5.32 (2.19–10.7), and 8.22 (3.84–13.8) kPa, respectively (Fig. 1). Liver stiffness augmented with the increment in the fibrosis stage, and we observed a significant difference in each intergroup comparison.

### Diagnostic accuracy of noninvasive markers for estimating advanced fibrosis (stages 3–4)

**Figure 2:**
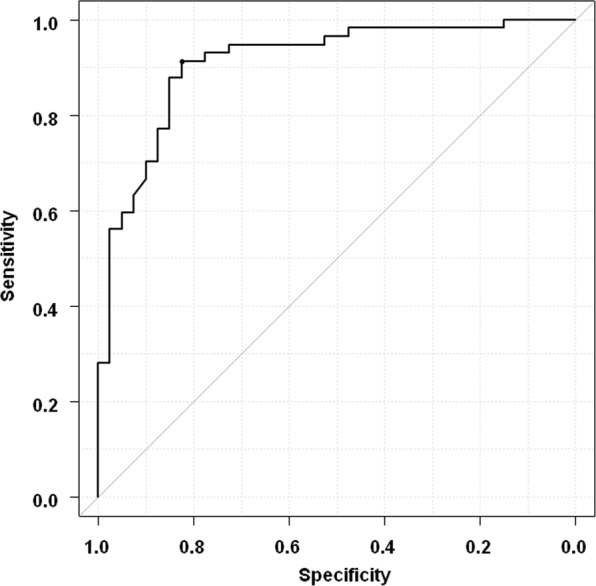
The prediction of advanced fibrosis (stages 3–4) by magnetic resonance elastography (MRE). The receiver operating characteristics curve of MRE for advanced fibrosis.

We predicted advanced fibrosis (stages 3–4) using liver stiffness by MRE. The area under the receiver operating characteristic (AUROC) value for the predictions was 0.910 (Fig. 2). Similarly, the AUROC values for predicting advanced fibrosis of *Wisteria floribunda* agglutinin-positive mac-2 binding protein (WFA^+^-M2BP), FIB-4, NAFLD fibrosis score (NFS), BARD score, platelet counts, and aspartate aminotransferase to platelet ratio index (APRI) were 0.841, 0.836, 0.831, 0.685, 0.799, and 0.780, and the AUROC was higher in MRE than serum fibrosis markers. The optimal cutoff value of liver stiffness for advanced fibrosis was 4.07 kPa; the sensitivity, specificity, positive predictive value (PPV), and negative predictive value (NPV) were 91.2%, 82.5%, 88.1%, and 86.8%, respectively.

### Estimation of severe liver stiffness by simple serum markers

**Figure 3:**
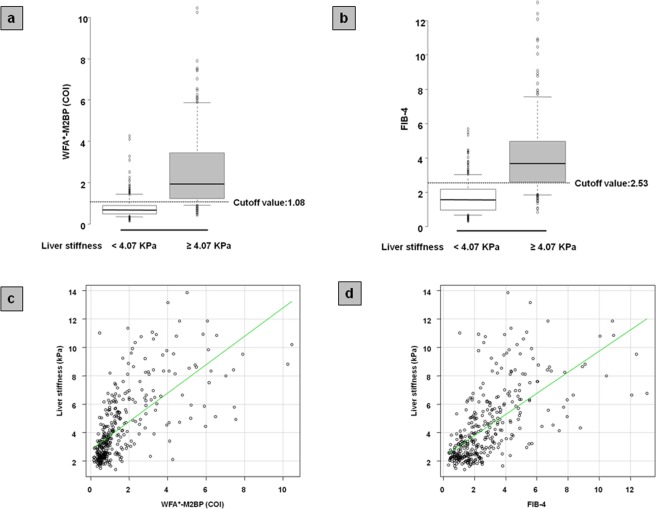
The correlation between liver stiffness and serum fibrosis markers. (**a**) The boxplot of WFA^+^-M2BP according to liver stiffness. (**b**) The boxplot of FIB-4 according to liver stiffness. The boxplot of serum fibrosis markers was shown according to liver stiffness. White box, patients with liver stiffness <4.07 kPa; gray box, patients with liver stiffness ≥4.07 kPa. The bottom and top of each box represent the 25^th^ and 75^th^ percentiles, giving the interquartile range. The line through the box indicates the median value, and the error bar indicates 10^th^ and 90^th^ percentiles. (**c**) The correlation between liver stiffness and WFA^+^-M2BP. (**d**) The correlation between liver stiffness and FIB-4.

Based on our findings, we defined severe liver stiffness as liver stiffness ≥4.07 kPa on MRE. We investigated the ability of serum fibrosis markers to predict severe liver stiffness. In the ROC analysis performed to estimate severe liver stiffness, the AUROC values of WFA^+^-M2BP, FIB-4, NFS, BARD score, platelet counts, and APRI were 0.897, 0.880, 0.862, 0.757, 0.827, and 0.804, respectively; based on these results, the following study was examined using WFA^+^-M2BP and FIB-4. The cutoff values for WFA^+^-M2BP and FIB-4 were 1.08 and 2.53, respectively (Table 2; Fig. 3a,b). The PPV and NPV of WFA^+^-M2BP were 78.8% and 88.3%, respectively, whereas those for FIB-4 were 77.4% and 84.0%, respectively. In addition, the correlation coefficient for the correlation between liver stiffness and WFA^+^-M2BP was 0.662 (Fig. 3c), exhibiting a significant correlation (*P* < 0.001). Furthermore, liver stiffness and FIB-4 correlated significantly (correlation coefficient, 0.634; *P* < 0.001; Fig. 3d).

**Table 2:** Prediction of severe liver stiffness.

	Cutoff value	AUROC	Sensitivity	Specificity	PPV	NPV
WFA^+^-M2BP	1.08	0.897	84.2	84.0	78.8	88.3
FIB-4	2.53	0.880	77.4	84.0	77.4	84.0
NFS	−1.01	0.862	91.2	66.7	71.8	89.1
BARD score	3.00	0.757	63.2	78.7	73.7	69.4
Platelet counts	16.2	0.827	71.2	81.5	73.2	79.9
APRI	0.69	0.804	74.7	75.6	68.6	80.7

AUROC, area under the receiver operating characteristic curve; PPV, positive predictive value; NPV, negative predictive value; WFA^+^-M2BP, wisteria floribunda agglutinin-positive mac-2 binding protein; NFS, NAFLD fibrosis score; APRI, aspartate aminotransferase to platelet ratio index.

### The impact of age on diagnostic accuracy

**Figure 4:**
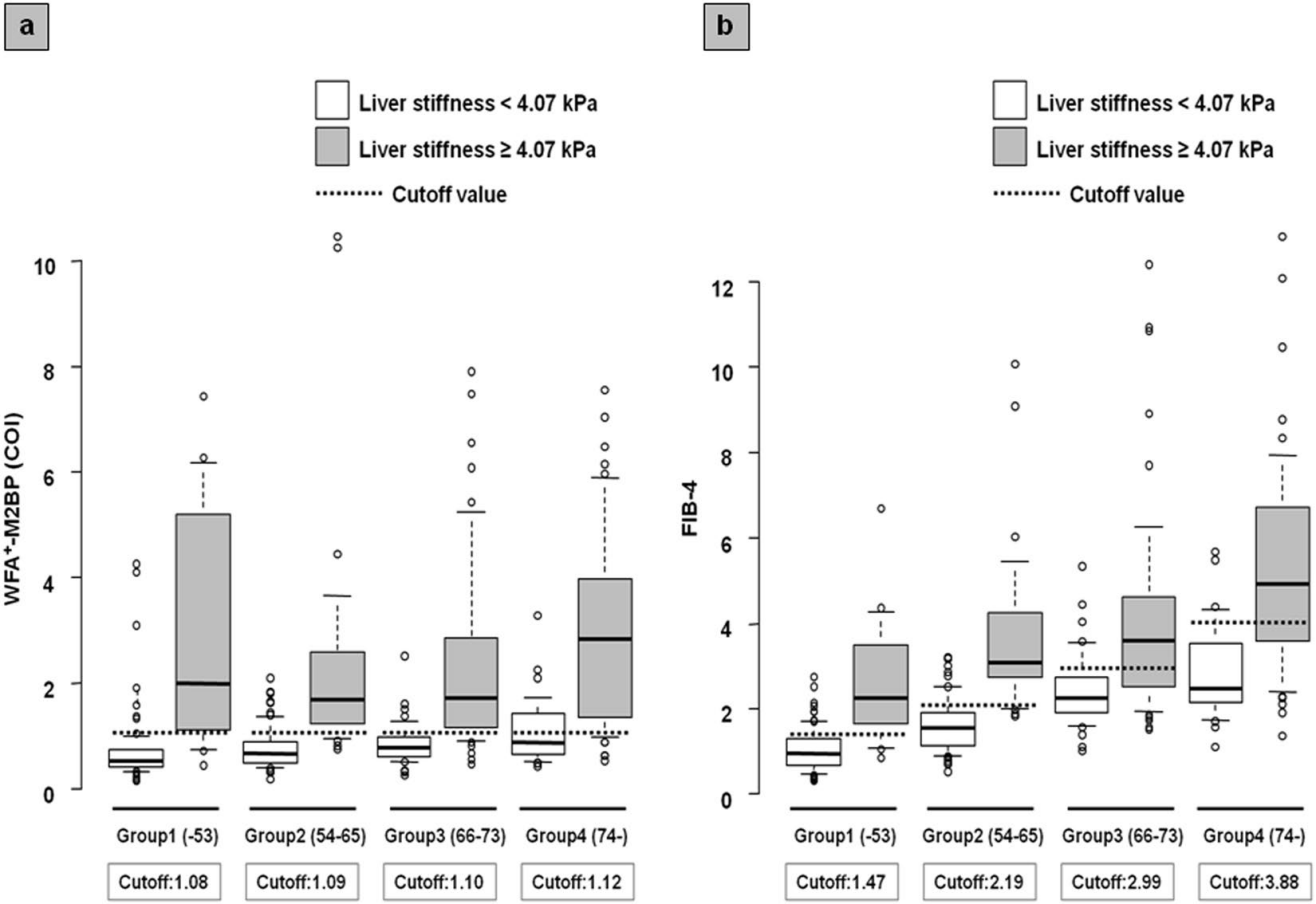
The boxplot of serum fibrosis markers in each age group according to liver stiffness. (**a**) The boxplot of WFA^+^-M2BP in each age group. (**b**) The boxplot of FIB-4 in each age group. The boxplot of serum fibrosis markers was shown according to liver stiffness. White box, patients with liver stiffness <4.07 kPa; gray box, patients with liver stiffness ≥4.07 kPa. The bottom and top of each box represent the 25^th^ and 75^th^ percentiles, giving the interquartile range. The line through the box indicates the median value, and the error bar indicates 10^th^ and 90^th^ percentiles.

We categorized all patients into four age groups (group 1: ≤53 years [n = 94]; group 2: 54–65 years [n = 91]; group 3: 66–73 years [n = 85]; group 4: ≥74 years [n = 82]) according to quartile and examined the impact of age on the diagnostic accuracy for WFA^+^-M2BP and FIB-4 to estimate severe liver stiffness. Figure 4a,b shows the boxplot of WFA^+^-M2BP and FIB-4 in each age group based on liver stiffness. Using the cutoff values of WFA^+^-M2BP (≥1.08 COI), the sensitivity to predict severe liver stiffness (≥4.07 kPa) in groups 1–4 were 82.4%, 85.7%, 82.0%, and 85.3%, respectively (Table 3). Conversely, using the cutoff values of FIB-4 (≥2.53), the sensitivity to predict severe liver stiffness in groups 1–4 were 47.1%, 82.1%, 76.0%, and 86.3%, respectively; the sensitivity decreased in group 1. Likewise, the specificity of severe liver stiffness in each group for WFA^+^-M2BP and FIB-4 were 90.9%/85.7%/80.0%/67.7% and 98.7%/90.5%/68.6%/51.6%, respectively; the specificity of FIB-4 deteriorated in the elderly group. When investigating the optimal cutoff values in each age group, the cutoff values of WFA^+^-M2BP for estimating severe liver stiffness in groups 1–4 were 1.09, 1.08, 1.10, and 1.12, respectively, with no differences in the values between groups (Table 3). However, the cutoff values for FIB-4 in groups 1–4 were 1.47, 2.19, 2.99, and 3.88, respectively, suggesting that the cutoff value elevated in parallel with age. When using this cutoff value, the sensitivity of FIB-4 in group 1 increased to 82.4%, and the specificity in group 3/4 increased to 80.0% and 83.9% and the diagnostic accuracy of FIB-4 improved by using this cutoff value according to age.

**Table 3:** Prediction of severe liver stiffness by age quartile.

	Group	Age	Cutoff value	Sensitivity	Specificity	PPV	NPV
WFA^+^-M2BP	group1	-53	1.08	82.4	90.9	66.7	95.9
	group2	54–65	1.08	85.7	85.7	72.7	93.1
	group3	66–73	1.08	82.0	80.0	85.4	75.7
	group4	74-	1.08	86.3	67.7	81.5	75.0
FIB-4	group1	-53	2.53	47.1	98.7	88.9	89.4
	group2	54–65	2.53	82.1	90.5	79.3	91.9
	group3	66–73	2.53	76.0	68.6	77.6	66.7
	group4	74-	2.53	86.3	51.6	74.6	69.6
WFA^+^-M2BP	group1	-53	1.09	82.4	90.9	66.7	95.9
	group2	54–65	1.08	85.7	85.7	72.7	93.1
	group3	66–73	1.10	80.0	85.7	88.9	75.0
	group4	74-	1.12	86.3	71.0	83.0	75.9
FIB-4	group1	-53	1.47	82.4	83.1	51.9	95.5
	group2	54–65	2.19	85.7	87.3	75.0	93.2
	group3	66–73	2.99	66.0	80.0	82.5	62.2
	group4	74-	3.88	72.5	83.9	88.1	65.0

PPV, positive predictive value; NPV, negative predictive value; WFA^+^-M2BP, wisteria floribunda agglutinin-positive mac-2 binding protein.

## Discussion

In this study, liver stiffness measured by MRE markedly correlated with the histological fibrosis stage in NAFLD. In addition, serum fibrosis markers, such as WFA^+^-M2BP and FIB-4, exhibited high predictive accuracy for severe liver stiffness. In particular, the diagnostic accuracy of WFA^+^-M2BP was not affected by age, and WFA^+^-M2BP could be used to identify patients with NAFLD who should be examined by MRE for diagnosing advanced fibrosis. Furthermore, identifying high-risk cases using WFA^+^-M2BP from a large number of patients with NAFLD has clinical significance.

This study aimed to predict advanced fibrosis based on MRE stiffness. Regarding the comparison of the pathological findings, this study validated the high accuracy of MRE for estimating advanced fibrosis, as reported previously^15^. Considering the increase in the number of elderly patients with complications, it is challenging to perform liver biopsy for all cases. In addition, liver biopsy involves several diagnostic discrepancies among examiners and correlates with poor diagnostic objectivity^16^. MRE is considered the most precise modality to replace liver biopsy and is currently being used in clinical trials instead of liver biopsy to assess the treatment effects^12,13^.

The major limitation of MRE is its high cost, which makes using this method for screening and targeting all patients with NAFLD challenging. Thus, we aimed to enclose high-risk patients with NAFLD who should undergo MRE using serum fibrosis markers. WFA^+^-M2BP is a serum fibrosis marker, and we have previously reported its efficacy in estimating fibrosis and HCC development in viral hepatitis^17–19^; its utility for the prediction of fibrosis and HCC development has also been reported in NAFLD^20–23^. This study illustrated that WFA^+^-M2BP has a high diagnostic accuracy for severe liver stiffness. The optimal cutoff value of WFA^+^-M2BP for estimating severe liver stiffness in this study was 1.08, which is within the range (WFA^+^-M2BP: 0.83–1.23) reported for the estimation of advanced fibrosis (histological stage 3–4) in previous studies. Although several reports have compared liver biopsy findings and WFA^+^-M2BP, to the best of our knowledge, this study is the first to compare MRE stiffness and WFA^+^-M2BP. The diagnosis using noninvasive and objective MRE could be extensively performed in the clinical practice of NAFLD^15^; thus, we believe that our finding regarding the efficacy of WFA^+^-M2BP to identify NAFLD patients who should be examined by MRE for advanced fibrosis is significant.

Reportedly, FIB-4 is useful for predicting fibrosis, HCC, and the development of complications in viral hepatitis and NAFLD^24–26^. Likewise, in this study, FIB-4 displayed a high diagnostic accuracy for severe liver stiffness. Nevertheless, the diagnostic accuracy of FIB-4 for fibrosis in hepatitis C or NAFLD reportedly changes with age^27,28^, and the optimal cutoff value is proposed to be set individually in different age groups. Hence, we assessed whether age affected the diagnostic accuracy of WFA^+^-M2BP and FIB-4 for the estimation of severe liver stiffness. In this study, the optimal cutoff value for WFA^+^-M2BP did not change with age; however, the optimal cutoff value for FIB-4 increased in parallel with age, which could be a major limitation of FIB-4 in the clinical practice. Although the diagnostic accuracy of both WFA^+^-M2BP and FIB-4 for severe liver stiffness were comparable as a whole, WFA^+^-M2BP is more suitable for identifying high-risk patients from a large number of patients with NAFLD because the cutoff values of WFA^+^-M2BP do not change with age.

Aging is a factor related to HCC development and prognosis in NAFLD, and FIB-4 including age is useful for these predictions^29^. The relationship between WFA^+^-M2BP and HCC development or prognosis has not been verified, and it is for further study whether WFA^+^-M2BP is more useful than FIB-4 in these predictions.

This study has several limitations. Being a hospital-based study, a selection bias might exist toward patients with suspected fibrosis. In fact, there were few patients with mild fibrosis. Hence, further population-based studies are warranted to confirm the efficacy of WFA^+^-M2BP to identify advanced fibrosis cases from a large number of NAFLD cases. The prediction accuracy of WFA^+^-M2BP in elderly patients was slightly reduced. WFA^+^-M2BP had a weak correlation with age, which may have reduced the prediction accuracy in elderly patients. The prediction accuracy of fibrosis using WFA^+^-M2BP in elderly people needs to be further examined.

In conclusion, WFA^+^-M2BP was useful in estimating severe liver stiffness in NAFLD patients with single cutoff value independent of age. Perhaps, the use of WFA^+^-M2BP for identifying high-risk patients from a large number of patients with NAFLD and determine patients who should be examined by MRE for advanced fibrosis has considerable clinical significance.

## Methods

### Patients

In this retrospective cross-sectional study, we assessed the utility of serum makers in estimating advanced fibrosis in patients with NAFLD. From April 2015 to October 2018, we enrolled 352 patients who fulfilled the following criteria: (1) the presence of hepatic steatosis on either imaging or liver histology; (2) daily ethanol consumption <20 g; (3) no infection with hepatitis B or C virus; and (4) no complications of autoimmune hepatitis or primary biliary cholangitis. All patients underwent MRE, and liver stiffness was assessed. Written informed consent was obtained from each patient. This study protocol conformed to the ethical guidelines in the Declaration of Helsinki and was approved by Musashino Red Cross Hospital Clinical Research Ethics Committee.

### Magnetic resonance elastography

MRE was performed using a 1.5-T magnetic resonance system with a superconducting magnet (SignaExcite HD MR 1.5T; GE Medical Systems, Milwaukee, WI) per the methods reported previously^8^. All the processing steps were automatic, without manual intervention, and yielded quantitative images of tissue shear stiffness in kPa. During the liver stiffness measurement, we avoided structures, such as large blood vessels and the gallbladder, on the constructed liver stiffness map, and set an ROI as large as possible to the measurable part. In addition, we measured liver stiffness in three slices and used the average value for data analyses.

### Clinical and laboratory data

We recorded patients’ age, sex, height, and weight during the liver stiffness measurement. In addition, fasting blood counts and biochemical tests were performed within 3 months before or after MRE using standard methods. We evaluated the body mass index using the following formula: weight (kg)/height^2^ (m). Notably, diabetes was diagnosed at a fasting blood sugar level of ≥126 mg/dL, at a random blood sugar level of ≥200 mg/dL and glycosylated hemoglobin level of ≥6.5%, or based on current treatment with antidiabetic drugs. Furthermore, the FIB-4 index^30^, NAFLD fibrosis score^31^, BARD score^32^, and APRI^33^ were evaluated for each patient.

### Histological evaluation

We performed a liver biopsy in 97 patients within 6 months before or after MRE. All liver biopsy specimens were laparoscopically obtained using 13G needles or through percutaneous ultrasound-guided liver biopsy using 15G needles. All specimens were fixed, paraffin-embedded, and stained using hematoxylin–eosin and Masson’s trichrome. A biopsy sample with a minimum size of 15-mm was required for diagnosis. Two senior pathologists who were blinded to the clinical data independently evaluated all the liver biopsy samples. We defined hepatic fibrosis (stage) as stage 1, zone 3 perisinusoidal fibrosis; stage 2, zone 3 perisinusoidal fibrosis with portal fibrosis; stage 3, zone 3 perisinusoidal fibrosis and portal fibrosis with bridging fibrosis; or stage 4, cirrhosis^34,35^.

### Statistical analyses

We analyzed the correlation between histological fibrosis stages and liver stiffness using the Mann–Whitney *U*-test. Using the ROC curves, we determined the optimal cutoff value for estimating advanced fibrosis and severe liver stiffness. The optimal cutoff value was ascertained using the Youden Index. In addition, we set statistical significance at *P* < 0.05. All statistical analyses were performed using EZR (Saitama Medical Center, Jichi Medical University, Saitama, Japan)^36^ and a graphical user interface for R (The R Foundation for Statistical Computing, Vienna, Austria).

## References

[1] Younossi ZM (2016). Global epidemiology of nonalcoholic fatty liver disease-Meta-analytic assessment of prevalence, incidence, and outcomes. Hepatology.

[2] Tanaka K (2018). Epidemiological survey of hemoglobin A1c and liver fibrosis in a general population with non-alcoholic fatty liver disease. Hepatol Res.

[3] Angulo P (2015). Liver Fibrosis, but No Other Histologic Features, Is Associated With Long-term Outcomes of Patients With Nonalcoholic Fatty Liver Disease. Gastroenterology.

[4] Dulai PS (2017). Increased risk of mortality by fibrosis stage in nonalcoholic fatty liver disease: Systematic review and meta-analysis. Hepatology.

[5] Vilar-Gomez E (2018). Fibrosis Severity as a Determinant of Cause-Specific Mortality in Patients With Advanced Nonalcoholic Fatty Liver Disease: A Multi-National Cohort Study. Gastroenterology.

[6] Angulo P (2002). Nonalcoholic fatty liver disease. N Engl J Med.

[7] Rockey DC, Caldwell SH, Goodman ZD, Nelson RC, Smith AD (2009). Liver biopsy. Hepatology.

[8] Yin M (2007). Assessment of hepatic fibrosis with magnetic resonance elastography. Clin Gastroenterol Hepatol.

[9] Higuchi M (2018). Prediction of hepatocellular carcinoma after sustained virological responses using magnetic resonance elastography. Clin Gastroenterol Hepatol.

[10] Imajo K (2016). Magnetic Resonance Imaging More Accurately Classifies Steatosis and Fibrosis in Patients With Nonalcoholic Fatty Liver Disease Than Transient Elastography. Gastroenterology.

[11] Park CC (2017). Magnetic Resonance Elastography vs Transient Elastography in Detection of Fibrosis and Noninvasive Measurement of Steatosis in Patients With Biopsy-Proven Nonalcoholic Fatty Liver Disease. Gastroenterology.

[12] Loomba R (2015). Ezetimibe for the treatment of nonalcoholic steatohepatitis: assessment by novel magnetic resonance imaging and magnetic resonance elastography in a randomized trial (MOZART trial). Hepatology.

[13] Harrison SA (2018). NGM282 for treatment of non-alcoholic steatohepatitis: a multicentre, randomised, double-blind, placebo-controlled, phase 2 trial. Lancet.

[14] Yoneda M (2018). Clinical strategy of diagnosing and following patients with nonalcoholic fatty liver disease based on invasive and noninvasive methods. J Gastroenterol.

[15] Loomba R (2018). Role of imaging-based biomarkers in NAFLD: Recent advances in clinical application and future research directions. J Hepatol.

[16] Pournik O (2014). Inter-observer and Intra-observer Agreement in Pathological Evaluation of Non-alcoholic Fatty Liver Disease Suspected Liver Biopsies. Hepat Mon.

[17] Kuno A (2013). A serum “sweet-doughnut” protein facilitates fibrosis evaluation and therapy assessment in patients with viral hepatitis. Sci Rep.

[18] Tamaki N (2015). Wisteria floribunda agglutinin positive human Mac-2-binding protein as a predictor of hepatocellular carcinoma development in chronic hepatitis C patients. Hepatol Res.

[19] Yasui Y (2018). Wisteria floribunda agglutinin-positive Mac-2 binding protein predicts early occurrence of hepatocellular carcinoma after sustained virologic response by direct-acting antivirals for hepatitis C virus. Hepatol Res.

[20] Abe M (2015). Association between Wisteria floribunda agglutinin-positive Mac-2 binding protein and the fibrosis stage of non-alcoholic fatty liver disease. J Gastroenterol.

[21] Atsukawa M (2018). Serum Wisteria floribunda agglutinin-positive Mac-2 binding protein more reliably distinguishes liver fibrosis stages in non-alcoholic fatty liver disease than serum Mac-2 binding protein. Hepatol Res.

[22] Kawanaka M (2018). Wisteria floribunda agglutinin-positive Mac-2 binding protein predicts the development of hepatocellular carcinoma in patients with non-alcoholic fatty liver disease. Hepatol Res.

[23] Ogawa Y (2018). Wisteria floribunda agglutinin-positive Mac-2-binding protein and type 4 collagen 7S: useful markers for the diagnosis of significant fibrosis in patients with non-alcoholic fatty liver disease. J Gastroenterol Hepatol.

[24] Tamaki N (2013). Noninvasive estimation of fibrosis progression overtime using the FIB-4 index in chronic hepatitis C. J Viral Hepat.

[25] Tamaki N (2014). Non-invasive prediction of hepatocellular carcinoma development using serum fibrosis marker in chronic hepatitis C patients. J Gastroenterol.

[26] Takahashi Y (2015). Non-alcoholic fatty liver disease fibrosis score and FIB-4 scoring system could identify patients at risk of systemic complications. Hepatol Res.

[27] Ishiba H (2018). The novel cutoff points for the FIB4 index categorized by age increase the diagnostic accuracy in NAFLD: a multi-center study. J Gastroenterol.

[28] Kurosaki, M. & Izumi, N. External validation of FIB-4: diagnostic accuracy is limited in elderly populations. *Hepatology* **47**, 352; author reply 352–353 (2008). 10.1002/hep.21978 18161729

[29] Ito T (2019). Utility and limitations of noninvasive fibrosis markers for predicting prognosis in biopsy-proven Japanese non-alcoholic fatty liver disease patients. J Gastroenterol Hepatol.

[30] Sterling RK (2006). Development of a simple noninvasive index to predict significant fibrosis in patients with HIV/HCV coinfection. Hepatology.

[31] Angulo P (2007). The NAFLD fibrosis score: a noninvasive system that identifies liver fibrosis in patients with NAFLD. Hepatology.

[32] Harrison SA, Oliver D, Arnold HL, Gogia S, Neuschwander-Tetri BA (2008). Development and validation of a simple NAFLD clinical scoring system for identifying patients without advanced disease. Gut.

[33] Wai CT (2003). A simple noninvasive index can predict both significant fibrosis and cirrhosis in patients with chronic hepatitis C. Hepatology.

[34] Kleiner DE (2005). Design and validation of a histological scoring system for nonalcoholic fatty liver disease. Hepatology.

[35] Pathological Findings of NASH and NAFLD: for Guidebook of NASH and NAFLD, 2015: The Japan Society of Hepatology. *Hepatol Res* **47**, 3–10 (2017). 10.1111/hepr.12847 27889947

[36] Kanda Y (2013). Investigation of the freely available easy-to-use software ‘EZR’ for medical statistics. Bone Marrow Transplant.

